# Erratum: Ubiquitous overexpression of the DNA repair factor *dPrp19* reduces DNA damage and extends *Drosophila* life span

**DOI:** 10.1038/s41514-017-0008-9

**Published:** 2017-08-15

**Authors:** Kathrin Garschall, Hanna Dellago, Martina Gáliková, Markus Schosserer, Thomas Flatt, Johannes Grillari

**Affiliations:** 1 Department of Ecology and Evolution, University of Lausanne, Lausanne, Switzerland; 2 Department of Biotechnology, BOKU – University of Natural Resources and Life Sciences, Vienna, Vienna, Austria; 3 Institut für Populationsgenetik, Vetmeduni Vienna, Vienna Austria; 4 Department of Developmental Molecular Biology, Max Planck Institute for Biophysical Chemistry, Göttingen, Germany; 5 Christian Doppler Laboratory on Biotechnology of Skin Aging, Dept. of Biotechnology, BOKU – University of Natural Resources and Life Sciences, Vienna, Vienna, Austria; 6 Evercyte GmbH, Vienna, Austria

**Erratum to:** *npj Aging and Mechanisms of Disease* (2017); doi:10.1038/s41514-017-0005-z; Published 15 August 2017

After online publication of this article, the authors noticed an error in Fig. 2.

**Fig. 2:**
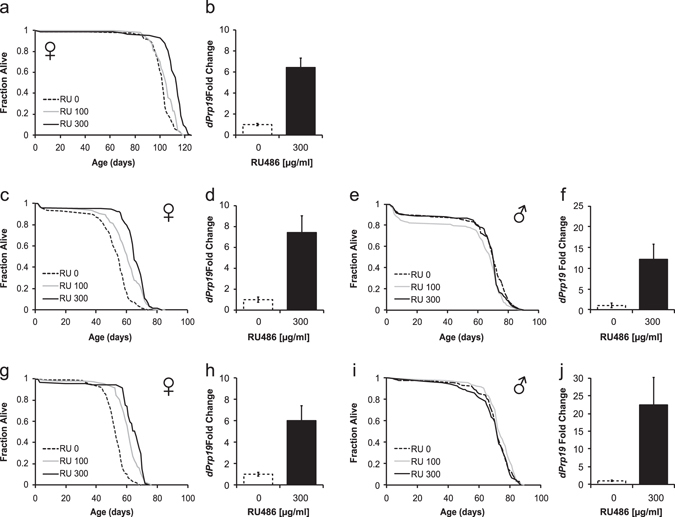
Overexpression of *dPrp19* leads to dose-dependent extension of female but not male life span. Effects of the induction of the *dPrp19* UAS cassette in three independent chromosomal insertions of the same transgenic construct on adult survival and *dPrp19* mRNA levels: **a**, **b** *Tub*GS-Gal4 > UAS-*dPrp19-1* (females only), **c–f** *da*GS-Gal4 > UAS-*dPrp19-2*(females and males), and **g–j** *da*GS-Gal4 > UAS-*dPrp19-4* (females and males). **a**, **c**, **e**, **g**, and **i** show survival curves of experimental flies at three concentrations of the inducer drug RU486; **b**, **d**, **f**, **h**, and **j** show quantification of *dPrp19* expression levels relative to the *Rp49* control after 72 h of exposure to 300 µg/ml RU486. For all three overexpression constructs, we find a significant dose-dependent extension of female life span. Overall, we did not find any life span extension in males (for *da*GS-Gal4 > UAS-*dPrp19*-2 males at 100 µg/ml RU486 we observed a slight reduction in survival, possibly due to inadvertent ‘‘setup mortality” that might have occurred when the assay was set-up). For details of life span statistics see Supplementary Table 1 (females) and Supplementary Table 2 (males); for experimental details see Materials and Methods

In panels **a**, **c**, and **g** of Fig. 2, there are the *y*-axes missing. The correct version of this figure appears below as Fig. 2.

This has now been corrected in the HTML and PDF versions of this Article.

The authors apologize for any inconvenience caused.

